# Estimation of the proper gap ratio using preoperative radiography for posterior tibial slope maintenance in biplanar open wedge high tibial osteotomy

**DOI:** 10.1186/s13018-023-03712-w

**Published:** 2023-03-20

**Authors:** Jung-Ro Yoon, Young Yoon Koh, Seung Hoon Lee

**Affiliations:** Department of Orthopedic Surgery, Veterans Health Service Medical Center, 53 Jinhwangdo-ro 61-gil, Gangdong-gu, Seoul, 05368 Korea

**Keywords:** High tibial osteotomy, Posterior tibial slope, Block

## Abstract

**Background:**

This study aimed to estimate the ratio of the anterior and posterior gaps before surgery that can minimize the posterior tibial slope (PTS) change through preoperative radiography, and to confirm whether the use of the block helps maintain the PTS during open wedge high tibial osteotomy (OWHTO).

**Methods:**

Patients who underwent OWHTO between 2015 and 2018 were included. To measure optimal anterior gap (AG) and posterior gap (PG) ratio, hinge to medial tibial tuberosity length (HTL), total osteotomy length (TOL), and PTS were measured using knee AP X-ray. Real AG and PG were measured using postoperative knee computed tomography. Use of the block was also confirmed.

**Results:**

Total 107 knees (95 patients) were included. The average ratio between HTL and TOL was 70.9%. The average ratio AG: PG was 72.9%. PTS increased significantly from 10.2° to 11.2° postoperatively (*p* = 0.006). When the difference in HTL: TOL and AG: PG, and the amount of PTS change were analyzed using linear regression, there was a statistically significant correlation (correlation coefficient: − 25.9; *p* < 0.001). There was no difference in AG: PG according to the use of the block (*p* = 0.882).

**Conclusion:**

In OWHTO, PTS change can be minimized by estimating the ratio of the AG and PG using radiographs, and is was approximately 70%. If the ratio is increased by 10% from the predicted value, the PTS increases by approximately 2.6°. Using a block during OWHTO did not have a considerable advantage in terms of PTS maintenance compared to the group not using a block.

***Level of evidence*:**

Level IV.

## Background

Open wedge high tibial osteotomy (OWHTO) is commonly used as the correction of the coronal plane can be adjusted through the difference in the opening gap when compared to close wedge high tibial osteotomy (CWHTO). However, several studies have reported that OWHTO can increase the posterior tibial slope (PTS) compared to CWHTO [1, 2]. To compensate for this defect, some studies recommend release of the posterior soft tissue, complete osteotomy of the posterior cortex of the tibia, and separate fixation between the anterior and posterior gaps [3, 4]. Several other studies have recommended a method of creating a difference between the anterior opening gap (AG) and posterior opening gaps (PG) and reported that the change in PTS can be sufficiently maintained using this method [5, 6].

**Fig. 1:**
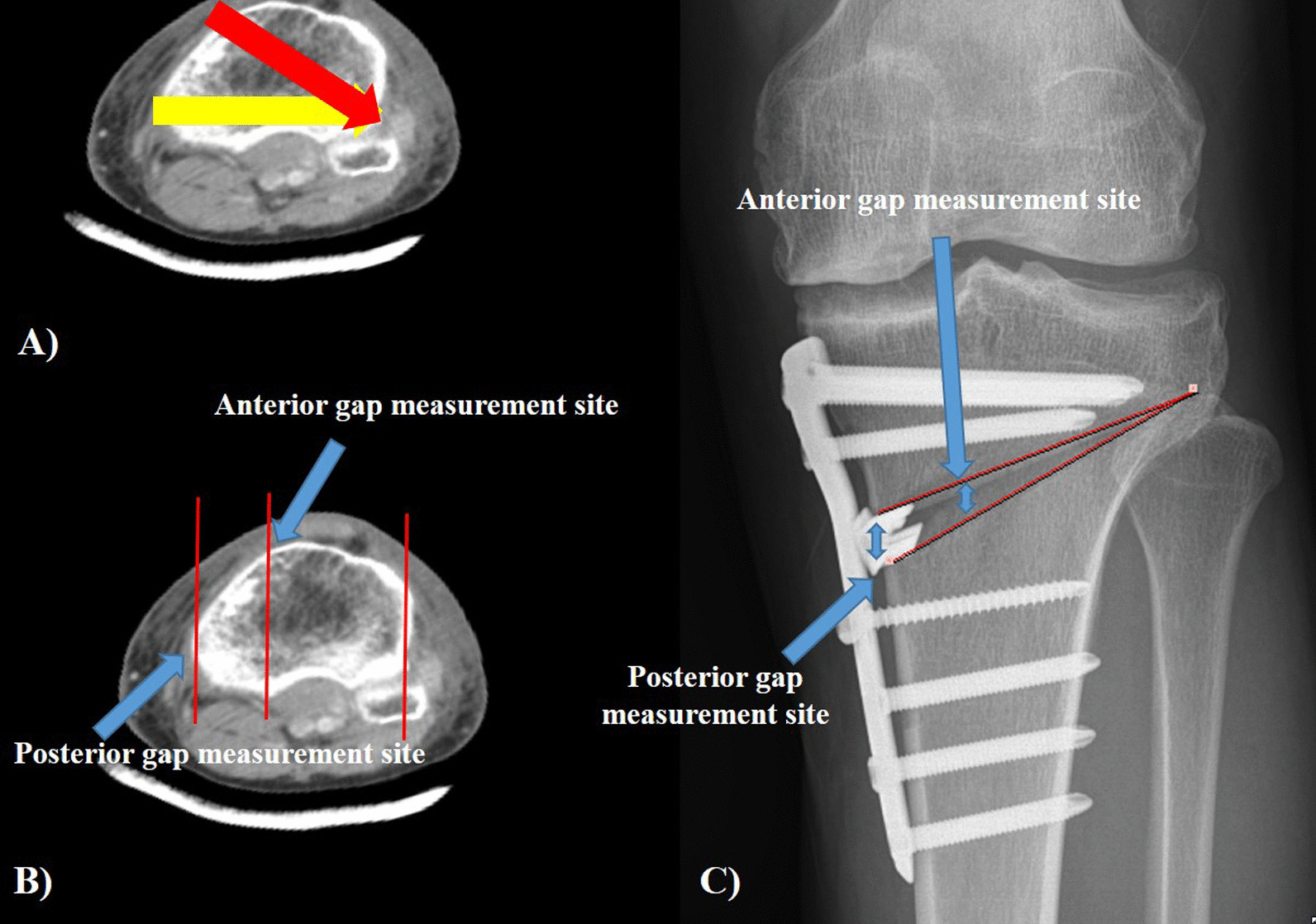
Reason for the difference between anterior gap and posterior gap. **a** Horizontal striped arrow: desired osteotomy direction, vertical striped arrow: real osteotomy direction, **b** The anterior gap (AG) and posterior gap (PG) are not measured in one plane, **c** AG should be smaller than PG, geometrically

The increase in PTS during OWTHO is due to the shape of the proximal tibia [1]. As the proximal tibia has a triangular shape and the surface on which the plate is fixed is oblique to the coronal plane, if the surgeon performs an OWHTO along this surface, the anterior portion of the osteotomy is lifted and PTS is increased (Fig. 1). Additionally, OWHTO involves correction in the coronal plane, and the position where AG and PG are measured is not on a single plane but on an oblique plane. Therefore, if the AG and PG are similar, the osteotomy direction will be posterolateral, leading to correction not only in the coronal plane, but also in the sagittal plane, which is not desired. An increase in the PTS can be prevented with a simple distraction at the most posterior gap [6]. Some studies recommend opening the anterior portion of the opening gap only by approximately 67% compared to the posterior portion during OWHTO [5, 7]. However, it is not clear to apply this to all patients, because the shape of tibia, and the angle of osteotomy is different for each patient. Additionally, in some cases, the PTS is artificially controlled in the anterior cruciate ligament or posterior cruciate ligament deficiency knee during OWHTO [8]. In this case, many studies induced changes in PTS by adjusting the ratio of AG and PG. However, it is not known how much the ratio must be adjusted to change the PTS [9, 10].

Additionally, some HTO plates use metal blocks to increase the initial stability and early weight-bearing during rehabilitation [11, 12]. Most HTO blocks have a tapered shape, and the anterior height of the block is lower than the posterior height; therefore, using block in OWHTO is useful to reduce PTS change [13]. However, there are no reports on how use of the tapered block affects PTS.

Given this background, we wanted to measure the ratio of AG and PG appropriate for OWHTO using preoperative radiography and considered that use of a block could reduce the change in PTS. This study aimed to estimate the ratio of the AG and PG before surgery, which can minimize the PTS change using preoperative radiography and to confirm whether the use of the block maintained the PTS. Our hypotheses was the following: (1) PTS change can be minimized by estimating the ratio of AG and PG using radiography and the PTS change can be predicted using this ratio, and (2) the use of block can prevent an increase in the PTS.

## Materials and methods

### Patient demographics

All patients undergoing OWHTO from January, 2015 to December, 2018 were retrospectively included. The exclusion criteria were as follows: (1) OWHTO using a fixation material other than a plate (Ohtofix anatomical locking metal block plate, Ohtomedical Co. Ltd., Goyang, Korea) in order to reduce bias due to the difference in the fixation device; (2) fixation failure owing to hinge fracture; (3) simultaneous femoral osteotomy; and (4) patients who underwent around knee bone surgery. Institutional Review Board approval was prospectively obtained before any analysis was performed (approval number: BOHUN 2021-01-019).

### Geometrical measurement

**Fig. 2:**
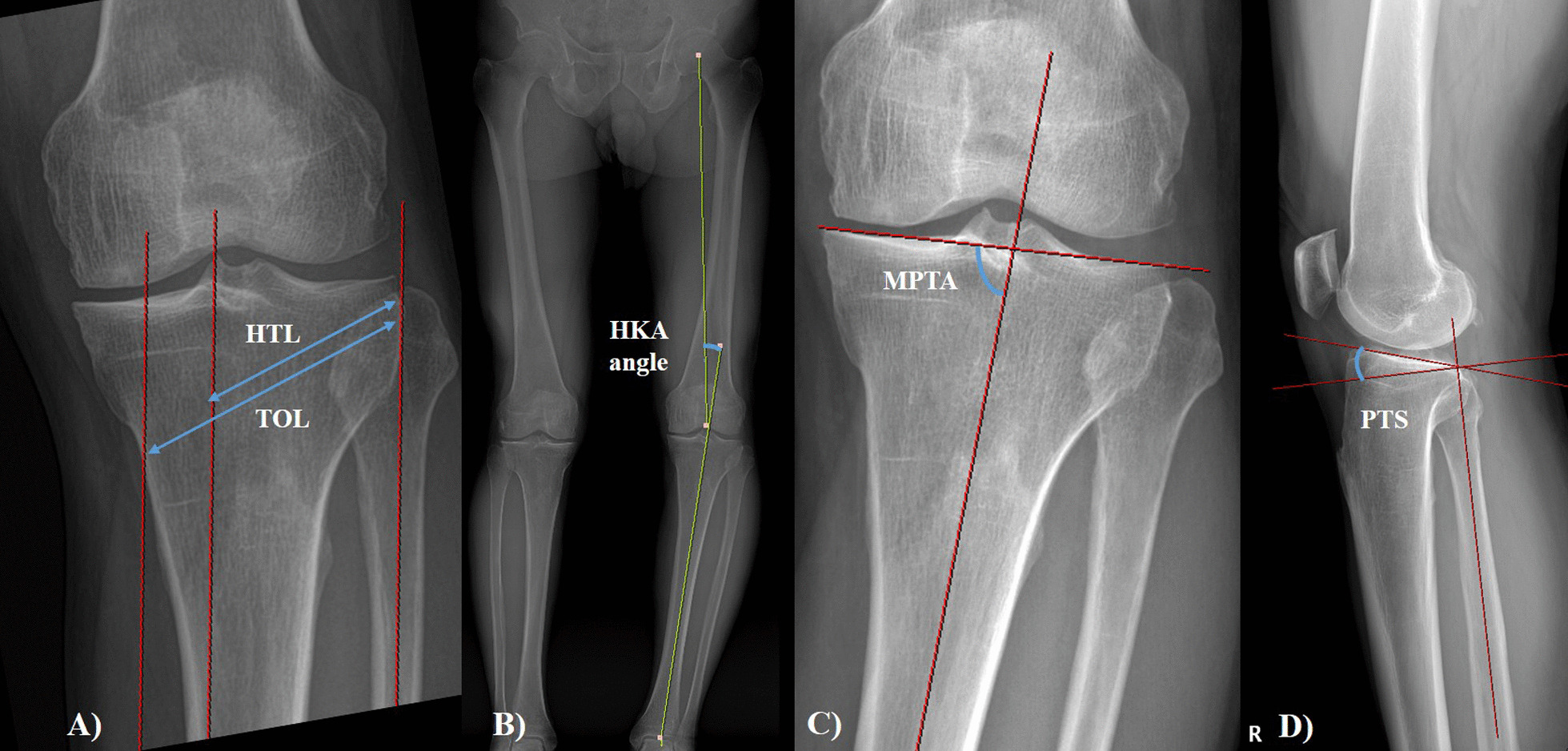
Geomterical measurement. **a** Hinge to tibial tuberosity length (HTL) and total osteotomy length (TOL) measurement, **b** hip-knee-ankle (HKA) angle, **c** medial proximal tibial angle (MPTA), **d** posterior tibial slope (PTS)

To make the coronal correction, the ratio of AG and PG in knee AP radiographs can be measured, as illustrated in Fig. 2. Geometrically, the ratio of AG and PG is equal to the ratio of the length from the hinge to the medial border of the tibial tuberosity (hinge to tibial tuberosity length, HTL) and the total osteotomy length (TOL) (Fig. 1). The tibial tuberosity medial border was assumed to be a line vertically down from the medial spine. HTL, where the location of the anterior opening gap is measured, was measured using the point where the osteotomy line met the line drawn parallel to the tibial shaft from the medial tibial spine. TOL was measured from the hinge to a point approximately 4 cm below the medial knee joint line (Fig. 2) [14].

**Fig. 3:**
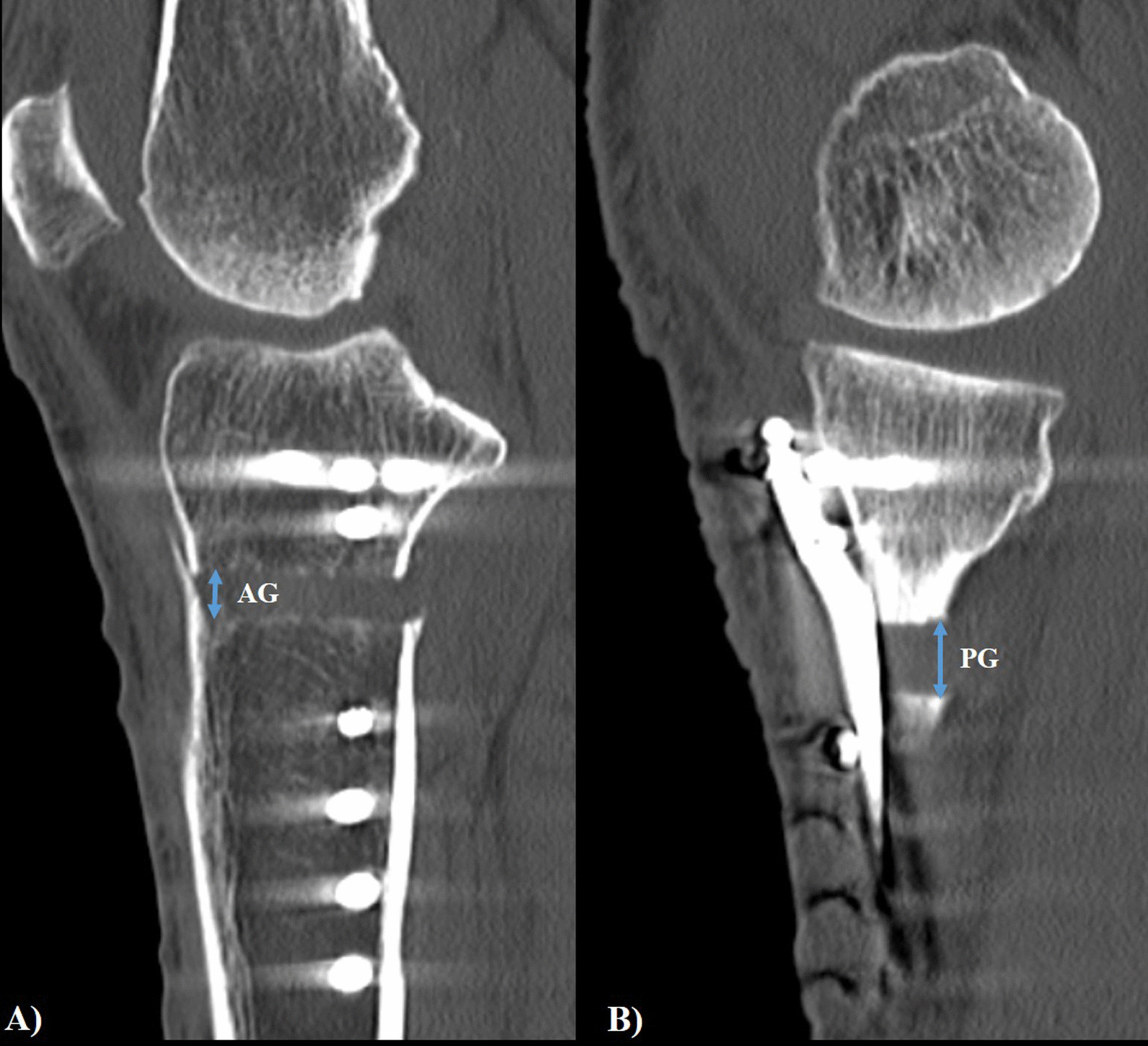
Gap measured on computed tomography (CT). **a** The anterior gap is measured at the most medial part of the tibial tubercle and **b** the posterior gap is measured at the most medial and posterior height of the osteotomy site using the CT sagittal view

PTS was measured using the angle between the fibular shaft and the medial tibial plateau line on the knee lateral radiographs before surgery, immediately after surgery, and 1 year after surgery [15]. The AG and PG of the osteotomy site were measured using computed tomography (CT) performed 1 week after surgery [16]. AG and PG were measured as the osteotomy gap at the medial border of the biplanar osteotomy site and the medial margin of the tibia, respectively using the knee sagittal CT view (Fig. 3). The medial proximal tibial angle (MPTA) was measured as the medial value at the angle between the line connecting the centers of the tibial metaphysis and diaphysis and the line connecting the tibial plateau [17]. The hip-knee-ankle (HKA) angle was also measured using a lower extremity long bone radiograph radiographs before surgery, immediately after surgery, and 1 year after surgery. The measurements were performed using the INFINITT picture archiving and communication system (INFINITT Healthcare, Seoul, South Korea). The reliability of angle measurements was assessed by examining the intra-rater and inter-rater reliability using the intra-class correlation coefficient (ICC).

### Surgical technique

The Miniaci method was used for preoperative planning, and the goal of correction was to have a weight-bearing line at the Fujisawa point [18]. An oblique incision was made at the medial proximal tibia area, and the pes anserine tendon was detached from the tibia [19, 20]. Subperiosteal elevation of the superficial medial collateral ligament (MCL) was also performed. Subsequently, the Hohmann retractor was inserted under the superficial MCL space, and the neurovascular structure was protected. Two Kirschner wires were inserted into the posterior cortex of the proximal tibia, which was considered as the hinge point. Horizontal osteotomy was performed approximately 1 cm medial to the lateral tibial cortex along the K-wire. After horizontal osteotomy, an additional anterior osteotomy was performed. Retrotubercular osteotomy was performed in a direction parallel to the patella tendon at the medial border of the tibial tubercle. The posterior cortex of the tibia was elevated to the planned length and fixed using an HTO locking plate. To maintain the PTS, tibia opening was performed in the tibia posterior cortex,, and the AG height of the osteotomy was kept at 60%–70% of the PG using caliper [5]. The use of block was determined by surgeon’s preference and block was positioned according to the tibia posterior cortex margin. After HTO, we did not allow weight-bearing until 3 weeks after HTO, allow partial weight-bearing until 6 weeks, and allow full weight-bearing. Range of motion exercise was not restricted postoperatively.

### Statistics

All statistical analyses were performed using the SPSS statistical package (Version 22.0, IBM Corp., Armonk, NY, USA). Linear regression was used to compare the gap ratio and PTS change, and an independent T-test was used to compare several angles before and after surgery. The difference in the change of PTS according to the use of the block was compared using independent T-test. All measurements on radiographs and CT were performed by an orthopedic surgeon (LSH) with no affiliation with the operation. The measurements were performed within a 2-week interval. Finally, the average of the two values was used for the analysis. Statistical significance was considered when the p-value was less than 0.05.

## Result

A total of 129 knees (115 patients) underwent OWHTO during the study period. A total of 107 knees (95 patients) were included in this study. Among them, twelve patients underwent bilateral OWHTO (Table 1). The mean age was 63.6 years, with 47 men and 48 women included in the study. There were three patients with Kellgren-Lawrence (KL) grade I, 33 with KL II, and 71 with KL III. The average body mass index was 26.4 (SD, 4.0).) The inter- and intra-observer reliabilities for the measurement of radiologic parameters were satisfactory and the mean values were 0.77 (ranging from 0.70 to 0.84) and 0.78 (ranging from 0.71 to 0.85), respectively.

1. Changes in posterior tibial slope according to the gap difference

**Table 1:** Demographics of included patients

	Block	No block	Total	*p* value
Number	49	58	107	
Age (year)	61.7 ± 7.9	65.2 ± 5.4	63.6 ± 6.9	0.065
Height (cm)	160.4 ± 9.2	159.2 ± 9.4	159.8 ± 9.3	0.991
Weight (kg)	69.4 ± 12.1	66.3 ± 13.3	67.7 ± 12.,8	0.657
BMI	26.9 ± 3.6	26.1 ± 4.3	26.4 ± 4.0	0.950
Initial Kellgren–Lawrence grade	I: 0	I: 3	I: 3	0.095
	II: 11	II: 22	II: 33	
	III: 38	III: 33	III: 71	

**Fig. 4:**
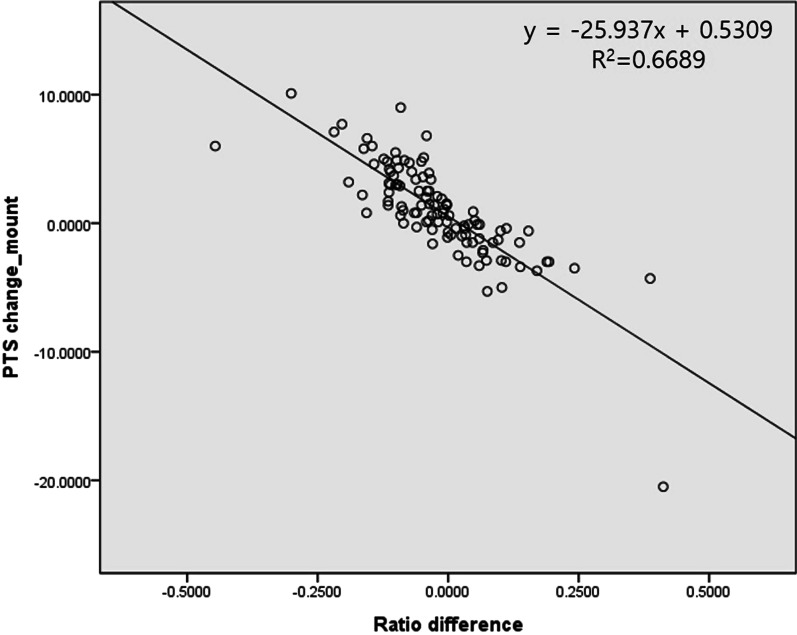
The difference in ratio between the hinge to tibial tuberosity length (HTL): total osteotomy length (TOL), anterior gap (AG): posterior gap (PG), and the amount of posterior tibial slope (PTS) changes are analyzed using linear regression analysis

The average length of the osteotomy measured using a knee AP radiograph before surgery was 69.5 mm and the average length from the hinge site to the medial tibial tubercle border was 49.3 mm. The mean ratio of this length was 70.9% (Table 2). The average AG and PG measured using CT after OWHTO were 6.8 mm and 9.4 mm, respectively, and the average AG: PG ratio was 72.9%. The PTS increased significantly from 10.2° preoperatively to 11.2° postoperatively (*p* = 0.006). When the difference in ratio between HTL: TOL and AG: PG and the amount of PTS change were analyzed using linear regression analysis, there was a statistically significant correlation with the correlation coefficient (− 25.9; *p* < 0.001), indicating that when the ratio difference is 10%, the PTS change is approximately 2.6° (Fig. 4).

2. Change in posterior tibial slope according to metal block use

**Table 2:** Estimated optimal anterior gap and posterior gap ratio to maintain posterior tibial slope using X-ray, and real posterior slope change after high tibial osteotomy

Estimated PTS change	
HTL (mm)	49.3 ± 5.7
TOL (mm)	69.5 ± 7.3
HTL: TOL ratio (optimal AG:PG ratio)	70.9 ± 3.5%
AG (mm)	6.8 ± 1.9
PG (mm)	9.4 ± 2.3
AG:PG ratio	72.9 ± 11.9%
Difference between HTL:TOL ratio and AG:PG ratio	1.9 ± 11.7%

Real parameter changes			
	Preoperative	Postoperative	p-value
MPTA (°)	85.1 ± 2.4	92.2 ± 2.74	< 0.001
HKA angle (°)	7.5 ± 2.8 (varus)	1.7 ± 2.4 (valgus)	< 0.001
PTS (°)	10.2 ± 2.8	11.2 ± 3.9	0.006

HKA: hip-knee-ankle, MPTA: medial proximal tibial angle, PTS: posterior tibial slope, AG: anterior gap, PG: posterior gap, HTL: hinge to medial tibial tuberosity length, TOL: total osteotomy length

Of the 107 cases, the group using a block included 49 patients and the group not using the block comprised 58 patients (Table 3). There was no difference in the preoperative HKA angle and the amount of HKA angle correction in the two groups. The HKA angle after surgery become more valgus in the group not using the block (*p* = 0.003). There was no difference between the two groups in preoperative MPTA, postoperative MPTA, and the amount of MPTA correction. In addition, the use of block did not affect the amount of PTS change (*p* = 0.517). The gap measured using CT was significantly higher for PG in the group without the block (*p* = 0.022); however, the actual AG: PG ratio did not differ between the two groups (*p* = 0.882).

**Table 3:** Changes in gap and correction amount before and after high tibia osteotomy according to block use

	Block (*n* = 49)	No block (*n* = 58)	*p* value
Preoperative HKA angle (°)	7.8 ± 2.6	7.2 ± 2.2	0.259
Immediate postoperative HKA angle (°)	− 0.9 ± 2.2	− 2.3 ± 2.3	0.003
Postoperative 1 year HKA angle (°)	− 0.7 ± 2.1	− 2.1 ± 2.1	0.002
Immediate HKA change (°)	8.7 ± 3.1	9.5 ± 3.7	0.254
HKA change after 1 year (°)	8.5 ± 3.0	9.3 ± 3.5	0.244
Preoperative MPTA (°)	85.3 ± 2.1	85.0 ± 2.6	0.478
Postoperative MPTA (°)	92.0 ± 2.6	92.4 ± 2.9	0.488
MPTA change (°)	6.7 ± 3.3	7.4 ± 3.2	0.270
Preoperative PTS (°)	10.3 ± 2.4	10.0 ± 3.1	0.606
Immediate postoperative PTS (°)	11.6 ± 3.4	10.8 ± 4.2	0.349
Postoperative 1 year PTS (°)	11.3 ± 3.0	10.6 ± 4.1	0.415
Immediate PTS change (°)	1.3 ± 3.0	0.8 ± 4.3	0.517
PTS change after 1 year(°)	1.0 ± 2.8	0.6 ± 3.9	0.434
Real AG (mm)	6.4 ± 1.6	7.1 ± 2.6	0.065
Real PG (mm)	8.8 ± 1.7	9.8 ± 2.6	0.022
AG:PG ratio	72.7 ± 10.3	73.0 ± 13.2	0.882

HKA: hip-knee-ankle, MPTA: medial proximal tibial angle, PTS: posterior tibial slope, AG: anterior gap, PG: posterior gap

## Discussion

The principal findings of this study were as follows: PTS change can be minimized by estimating the ratio of the AG and PG using radiographs, and is was approximately 70%. If the ratio was increased by 10% from the predicted value, the PTS increased by approximately 2.6°. The use of a tapered block had no advantage compared to not using a block for PTS maintenance.

There are reports that the PTS is increased after OWHTO, and to prevent this, it is recommended that AG is lesser than PG during OWHTO. Several studies have recommended the ratio of AG and PG as 60–70% [5, 7, 21]. However, since the shape of the tibia varies between individuals, there is a problem in applying it uniformly. Additionally, in cases of ligament deficiency, the PTS needs to be artificially changed [8]. However, there has been no research on the gap ratio necessary to obtain the desired PTS change. In our study, the PTS increased by approximately 0.5° if the ratio of AG and PG was applied according to the ratio of TOL and HTL measured using a radiograph. This number was reported to be statistically significant; however, considering the measurement error, there was approximately no change in the PTS. In addition, the correlation coefficient between the ratio difference and PTS change was approximately − 25.9, indicating that when the ratio difference is 10%, the PTS change is approximately 2.6°. Alternatively, if the ratio of AG and PG is increased by 10% compared to the ratio of TOL and HTL, the PTS increases by 2.6°. Using this method, the ratio of AG and PG can be adjusted to obtain the desired PTS correction. This indicates that if the ratio of AG and PG is 10% greater than the ratio of HTL and TOL, the PTS increases by approximately 2.6°.

When there was no difference between the AG:PG ratio and the HTL:TOL ratio, the change in PTS converged to almost 0. This means that the most appropriate AG:PG ratio equals the HTL:TOL ratio, so it is about 71%. In our study, the ratio of HTL to TOL was averaged at approximately 71%. This is not considerably different from previously reported values. In addition, considering that the opening gap is mostly around 10 mm, the error from the existing value is approximately 0.1 mm, which is clinically negligible. However, the concept mentioned in this study can be used for more accurate preoperative planning as the ratio of AG and PG can be estimated by considering the shape of the tibia, which differs between individuals. In addition, although the use of a metal block may affect initial rehabilitation, is did not affect varus recurrence and PTS maintenance 1 year after HTO in this study.

Some studies have reported that the use of metal blocks increases early stability and enables early weight-bearing [7, 11, 12]. These blocks are tapered to reduce the impact on the PTS change. In this study, metal blocks were used in a total of 49 cases and the PTS was slightly increased in the group using a block than in the group that did not use a block; however, the difference was not statistically significant. However, considering the standard deviation, the amount of PTS change tended to be more uniform in the group using the block. Therefore, if the AG was artificially reduced to 60%–70% of the PG, the PTS change could be reduced without the need for a block.

## Limitations

This study has several limitations. First, the study included only radiologic findings and not clinical outcomes. Therefore, clinical outcomes according to the PTS change are unknown. Second, we analyzed biplanar OWHTO in this study. The actual site of tibia tubercle osteotomy, level of tibia osteotomy, and the osteotomy depth were confirmed intraoperatively, and the preoperative plan could have been different; moreover, an error was estimated. Third, the biplanar osteotomy site level was estimated by the medial tibial spine of the knee X-ray. However, this may be different from the actual biplanar osteotomy site. Fourth, this was a retrospective study; therefore, blocks were not used randomly.

## Conclusion

In OWHTO, the ratio of AG to PG that can minimize the PTS change can be estimated using preoperative knee radiography. If the ratio is increased by 10% from the predicted value, the PTS increases by approximately 2.6°. Using a block during OWHTO did not have a considerable advantage in terms of PTS maintenance compared to the group not using a block.

## Data Availability

The datasets analyzed during the current study are available from the corresponding author on reasonable request.

## Abbreviations

AG: Anterior gap
CWHTO: Close wedge high tibia osteotomy
HKA: Hip-knee-ankle
HTL: Tibial tuberosity length
MCL: Medial collateral ligament
MPTA: Medial proximal tibial angle
OWHTO: Open wedge high tibia osteotomy
PG: Posterior gap
PTS: Posterior tibial slope
TOL: Total osteotomy length

## Declarations

### Consents for publication

All presented cases in this report had written consents for publication.
